# Parasitic modulation of host development by ubiquitin-independent protein degradation

**DOI:** 10.1016/j.cell.2021.08.029

**Published:** 2021-09-30

**Authors:** Weijie Huang, Allyson M. MacLean, Akiko Sugio, Abbas Maqbool, Marco Busscher, Shu-Ting Cho, Sophien Kamoun, Chih-Horng Kuo, Richard G.H. Immink, Saskia A. Hogenhout

**Affiliations:** 1 Department of Crop Genetics, John Innes Centre, Norwich Research Park, Norwich NR4 7UH, UK; 2 The Sainsbury Laboratory, University of East Anglia, Norwich Research Park, Norwich NR4 7UH, UK; 3 Laboratory of Molecular Biology, Wageningen University and Research, Wageningen 6708 PB, the Netherlands; 4 Plant Developmental Systems, Bioscience, Wageningen University and Research, Wageningen 6708 PB, the Netherlands; 5 Institute of Plant and Microbial Biology, Academia Sinica, Taipei 11529, Taiwan

**Keywords:** vasculature-colonizing bacteria, insect vectors, plant pathogen, developmental phase transition, zombie plant, ubiquitin-proteasome system, targeted protein degradation, phloem, mycoplasma, non-culturable bacteria

## Abstract

Certain obligate parasites induce complex and substantial phenotypic changes in their hosts in ways that favor their transmission to other trophic levels. However, the mechanisms underlying these changes remain largely unknown. Here we demonstrate how SAP05 protein effectors from insect-vectored plant pathogenic phytoplasmas take control of several plant developmental processes. These effectors simultaneously prolong the host lifespan and induce witches’ broom-like proliferations of leaf and sterile shoots, organs colonized by phytoplasmas and vectors. SAP05 acts by mediating the concurrent degradation of SPL and GATA developmental regulators via a process that relies on hijacking the plant ubiquitin receptor RPN10 independent of substrate ubiquitination. RPN10 is highly conserved among eukaryotes, but SAP05 does not bind insect vector RPN10. A two-amino-acid substitution within plant RPN10 generates a functional variant that is resistant to SAP05 activities. Therefore, one effector protein enables obligate parasitic phytoplasmas to induce a plethora of developmental phenotypes in their hosts.

## Highlights

• Phytoplasma SAP05 proteins bind plant SPL and GATA transcription factors and RPN10
• SAP05 mediates degradation of SPLs and GATAs in a ubiquitin-independent manner
• SAP05 decouples plant developmental transitions and induces witches’ broom symptoms
• Engineering of plant RPN10 confers resistance to SAP05 activities

## Graphical abstract

**Figure:**
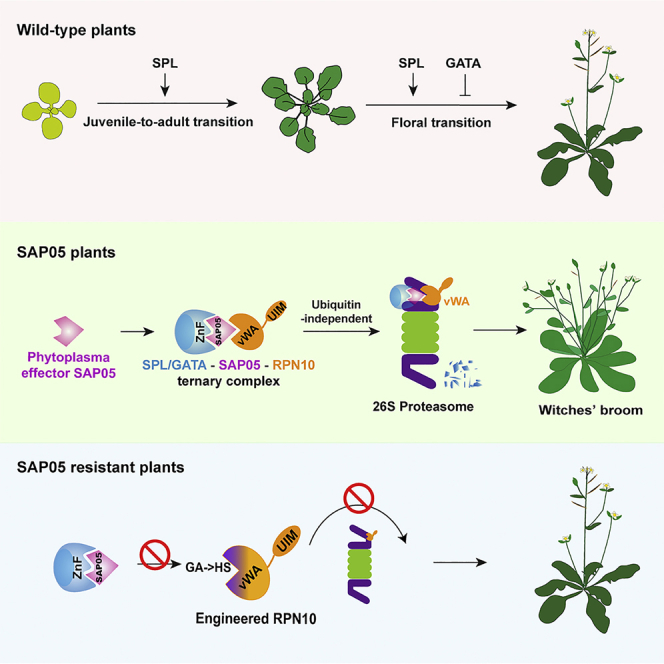

## Teaser

A virulence factor from an insect-vectored parasitic phytoplasma induces ubiquitin receptor-mediated developmental changes in the plant host that favor pathogenesis. This effector-receptor interaction can be modulated to engineer plants resistant to parasitic infection.

## Introduction

Parasites are known to modulate specific processes in hosts to promote colonization and virulence. Most parasites colonize one host, but a substantial number require multiple hosts to complete their life cycle. These parasites often depend on the hosts feeding on each other and, fascinatingly, appear to have evolved mechanisms to induce developmental and behavioral modifications in their hosts that increase the chance of interactions among host trophic levels (Hughes and Libersat, 2019; Le Fevre et al., 2015). For example, the trematode *Ribeiroia ondatrae* causes severe limb abnormalities in Pacific treefrogs, such as induction of extra limbs or aborting limbs, impairing movement of the frogs and increasing the risk of predation by birds, which are the definitive hosts of the trematode parasite (Johnson et al., 1999). These parasites are spectacular examples of how the reach of genes can extend beyond an organism to affect the surrounding environment, a phenomenon known as the extended phenotype (Dawkins, 1982). However, the molecular mechanisms underpinning these parasite-enforced host modifications are largely unknown, and there are ongoing debates about the extent to which these phenotypes are adaptive (Herbison et al., 2018; Johnson and Koshy, 2020).

One group of plant pathogens notorious for reprogramming host development consists of members of *Candidatus* (Ca.) Phytoplasma (Doi et al., 1967; The IRPCM Phytoplasma/Spiroplasma Working Team-Phytoplasma Taxonomy Group, 2004; Lee et al., 2000), which comprises a diverse genus of bacteria that cause global socioeconomically important insect-transmitted diseases (EPPO, 2021). Phytoplasmas infect most vascular plant species and often induce massive changes in plant architecture, such as excessive proliferation of shoots and branches (witches’ broom) and retrograde development of flowers into leaf-like organs (phyllody) (Hoshi et al., 2009; MacLean et al., 2011; Maejima et al., 2014; (Sugio et al., 2011a)). Notably, phytoplasmas are strict obligates that have a dual host cycle that alternates between plants (kingdom Plantae) and insects (kingdom Animalia) (Hogenhout et al., 2008). In plants, phytoplasmas colonize the cytoplasm of vascular phloem sieve cells that transport nutrients to growing plant tissues and spread systemically in plants via migration through the phloem cell sieve pores (Musetti et al., 2013). Sap-feeding insects that feed from the phloem, predominantly leafhoppers, planthoppers, and psyllids of the order Hemiptera, are often efficient phytoplasma vectors (Weintraub and Beanland, 2006). Phytoplasma-infected plants have been referred to as “zombie plants” because they exhibit extensive architectural changes, stop reproducing, and appear to serve solely as habitats for the phytoplasma pathogens and their insect vectors (Du Toit, 2014; MacLean et al., 2014; Orlovskis and Hogenhout, 2016; Rümpler et al., 2015). Phytoplasmas can be deleterious to their plant hosts, but they often have neutral or beneficial effects on their insect vectors, especially in established pathosystems where the bacteria and insects have co-evolved over long periods of time (Beanland et al., 2000; Malembic-Maher et al., 2020; Nault, 1990). The three-way interactions among phytoplasmas, plants, and insects are an excellent system to study the genetic basis of extended phenotypes created by obligate multi-host parasites (Huang et al., 2020; Sugio et al., 2011b).

Progress in the characterization of phytoplasma virulence factors is greatly accelerated by the ability of some phytoplasmas to colonize the model plant *Arabidopsis thaliana*. One of these phytoplasmas is Aster Yellows phytoplasma (AYP) strain Witches’ Broom (AY-WB; *Ca*. Phytoplasma *asteris*) (Hogenhout et al., 2008; Sugio et al., 2011b; Zhang et al., 2004). The main vector of AYPs in North America is the polyphagous aster leafhopper *Macrosteles quadrilineatus*, which migrates over long distances and transmits the bacteria to various crops, including oilseed rape, carrots, and several cereals (CABI, 2021; Frost et al., 2013a, 2013b). AYPs induce witches’ broom and phyllody symptoms and their occurrence can be high, sometimes contributing to loss of entire crop productions (Frost et al., 2013a). Phytoplasmas cause these symptoms by secreting proteins, known as effectors, that are unloaded from the phloem to adjacent plant tissues, such as shoot and apical meristems (Arashida et al., 2008; Bai et al., 2009; Hoshi et al., 2009; MacLean et al., 2011). Mining of the AY-WB genome for potential effectors resulted in identification of 56 candidate effector genes, called secreted AY-WB proteins (SAPs) (Bai et al., 2009). To date, only a few of these have been characterized. Among them, SAP11 binds and destabilizes *A. thaliana* TCP transcription factors, resulting in leaf shape changes and stem proliferation (Bai et al., 2009; Sugio et al., 2011a, 2014). SAP54 binds and degrades *A. thaliana* MADS box transcription factors by co-opting proteasome RAD23 shuttle factors, leading to development of leafy flowers (MacLean et al., 2011, 2014). Homologs of these effectors have been found in divergent phytoplasmas and shown to degrade TCPs and MADS box transcription factors of other plant species (Chang et al., 2018; Iwabuchi et al., 2020; Kitazawa et al., 2017; Lu et al., 2014; Maejima et al., 2014; Pecher et al., 2019; Tan et al., 2016; Wang et al., 2018). However, the phenotypes caused by SAP11, SAP54, and their homologs do not account for all of the extensive developmental phenotypes caused by phytoplasmas, such as prolonged lifespan and witches’ broom-type tissue proliferation other than stems.

Here we discovered that a phytoplasma effector, SAP05, binds and mediates degradation of multiple members of two distinct transcription factor families, the SPL family and the GATA family, leading to delayed plant aging and simultaneous proliferation of vegetative tissue and shoots. SAP05 mediates degradation through a ubiquitination-independent mechanism by co-opting the 26S ubiquitin receptor RPN10, which is highly conserved across eukaryotes. Remarkably, SAP05 does not bind the RPN10 of phytoplasma insect vectors, and only two RPN10 amino acids define binding specificity. We used this information to engineer a functional variant of plant RPN10 that mimics the insect RPN10 sequence. This mimic has lower affinity for SAP05 and confers plant resistance to SAP05 activity during phytoplasma infection. This work shows that one single phytoplasma effector co-opts one host proteasome protein to degrade multiple developmental regulators, inducing a plethora of adaptive phenotypic changes in their plant hosts.

## Results

### SAP05 alters plant architecture and reproduction

**Figure 1:**
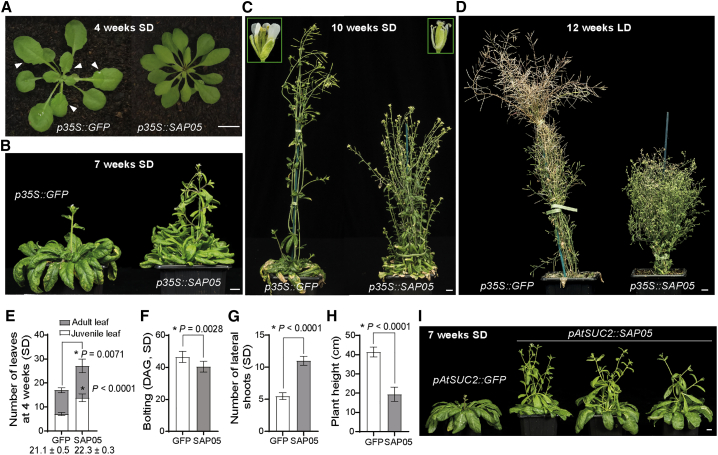
The phytoplasma effector SAP05 induces excessive shoot proliferation and sterility in *A. thaliana* (A–D) Representative images of transgenic plants expressing *SAP05* or *GFP* (control). Plants were grown under short-day (SD) or long-day (LD) conditions, and images were obtained 4 weeks (A), 7 weeks (B),10 weeks (C), and 12 weeks (D) after germination. Arrowheads in (A) indicate leaf serrations on GFP plants as opposed to the smoother leaf edges of SAP05 plants. Insets in (C) show enlarged images of mature flowers with the same magnification. Scale bars, 1 cm. For plants grown under LD, see also Figure S1. (E–H) Statistical analysis of phenotypes shown in (A)–(C): rosette leaf numbers of 4-week-old plants (E) and time of shoot emergence from rosettes (bolting time; F), number of shoots emerging from rosettes (lateral shoot number; G), and plant height (H) of 10-week-old SD plants. Numbers under the bars in (E) indicate the time when the first abaxial trichome appeared. DAG, day after germination. Data are mean ± SD; ^∗^p < 0.05, two-tailed Student’s t tests. (I) Morphology of a GFP control plant and three independent *A. thaliana* transgenic lines expressing SAP05 under control of the phloem-specific *AtSUC2* promoter. Images were obtained 7 weeks after germination. Scale bar, 1 cm.

**Figure S1:**
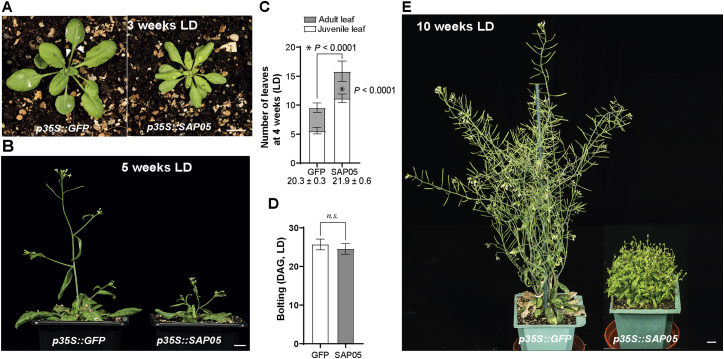
The morphology of SAP05-expressing At under LD conditions, related to Figure 1 (A-B) Representative images of plants stably producing SAP05 or GFP (control) grown under long-day (LD) conditions. Images were obtained at 3 weeks (A) and 5 weeks (B) after germination. Arrowheads in (A) indicate leaf serrations of GFP plants as opposed to the smoother leaf edges of SAP05 plants. Scale bars, 1 cm. (C-D) Statistical analysis of phenotypes shown in (A-B): numbers of rosette leave of 4-week-old plants (C) and time of shoot emergence from rosettes (bolting time; D). Numbers under the bars in (C) indicate the time (DAG) when the first abaxial trichome appeared. DAG, day after germination. Data are mean ± SD; ^∗^p < 0.05, two-tailed unpaired Student’s t tests. (E) A SAP05 plant exhibiting severe bushy and sterile phenotypes.

As part of functional screens with candidate AY-WB phytoplasma effectors, we found that SAP05 perturbs plant developmental processes by analyzing phenotypes of stable transgenic *A. thaliana* (At) lines that constitutively express the *SAP05* gene (without signal peptide) under control of the constitutive cauliflower mosaic virus (CaMV) *35S* promoter. These *SAP05*-expressing plants exhibited a range of architectural differences compared with control plants that express green fluorescent protein (GFP) from the same promoter (Figure 1; Figure S1). During vegetative growth, SAP05 plants displayed accelerated leaf initiations and produced more rosette leaves (Figures 1A and 1E; Figures S1A and S1C). In addition, the leaves of mature rosettes lacked serrated edges that are present in control plants (Figure 1A; Figure S1A). A closer examination revealed that the appearance of abaxial trichomes, which is characteristic of adult leaves, was also delayed in SAP05 plants (Figure 1E; Figure S1C). However, SAP05 plants initiated budding and flowering no later than the control plants under short-day (SD) and long-day (LD) conditions (Figure 1F; Figure S1D). SAP05 plants produced more lateral shoots and secondary branches, and these plants were reduced in height (Figures 1C, 1D, 1G, and 1H). At 12 weeks under LD conditions, GFP plants started to senesce, whereas SAP05 plants continued to grow (Figure 1D), suggesting that SAP05 delays plant senescence. Twenty-six of 32 independently transformed *p35S::SAP05* lines developed abnormal flowers and had greatly compromised fertility (Figure 1C, inset), with extremely bushy plants showing complete sterility (Figure S1E).

Because phytoplasmas secrete effector proteins into phloem cells, we generated stable transgenic *A. thaliana* lines expressing SAP05 from the phloem-specific *AtSUC2* promoter (Mathieu et al., 2007). *pAtSUC2::SAP05* plants exhibited similar architectural changes as *p35S::SAP05* plants, such as stunting, bushiness, and sterility (Figure 1I), phenotypes that resemble the witches’ broom symptoms typically observed in phytoplasma-infected plants.

### SAP05 destabilizes SPL and GATA plant transcription via the ubiquitin receptor RPN10

**Figure 2:**
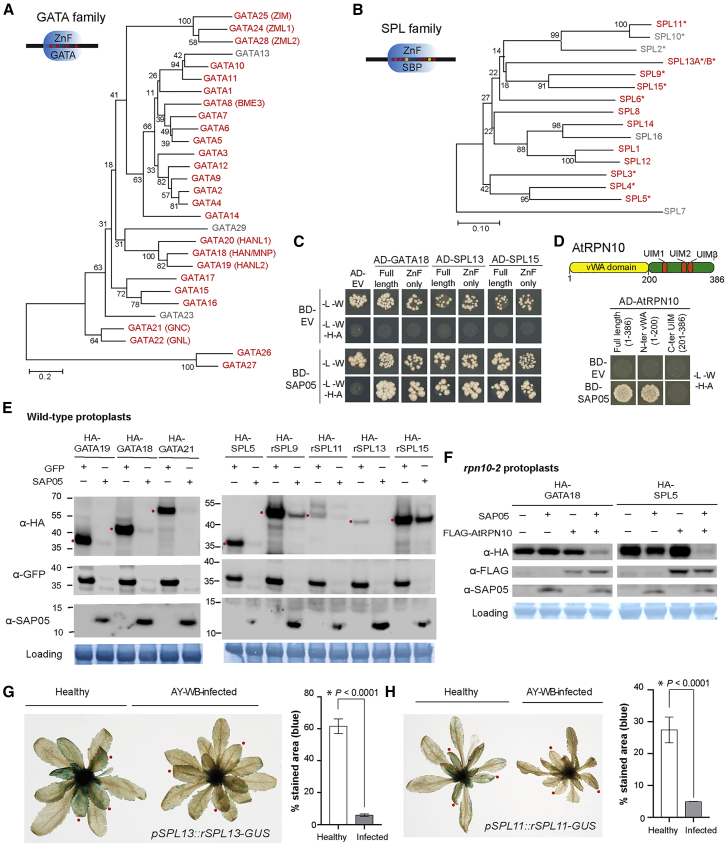
SAP05 destabilizes plant TFs of the SPL and GATA families via interaction with the ubiquitin receptor AtRPN10 **(**A and B**)** SAP05 interacts with most members of the *A. thaliana* GATA (A) and SPL (B) TFs families in Y2H assays. The phylogenies show SAP05 interactors in red and those that were not tested or had autoactivation activities in gray. Conserved zinc-finger (ZnF) domains are shown in the top left corners, with red and yellow dots indicating cysteine and histidine residues, respectively. SBP, SQUAMOSA promoter-binding protein; ^∗^, regulated by miR156. (C) SAP05 interacts with the ZnF domains of GATAs and SPLs in Y2H assays. EV, empty vector control. AD, GAL4-activation domain. BD, GAL4-DNA binding domain. (D) SAP05 interacts with AtRPN10 in Y2H assays. Top panel: graphical representation of AtRPN10 domains, with locations indicated in amino acids underneath. See Figure S6A for yeast growth on -L-W (lacking leucine and tryptophan) medium. (E) Western blot analysis of SAP05-mediated degradation of GATA and SPL proteins in protoplasts from wild-type *A. thaliana*. GFP, control; HA, hemagglutinin; rSPL, miR156-resistant form. Numbers on the left indicate molecular weight markers in kilodaltons. Red dots on the left of the blots indicate the expected sizes of the transiently expressed proteins. Protein loading was visualized using amido black staining. (F) Western blot analysis of SAP05 degradation assays in *rpn10-2* protoplasts. (G and H) GUS staining produced by the GUS fusions of the miR156-resistant forms of SPL11 (rSPL11) and SPL13 (rSPL13) in the transgenic *A. thaliana* is reduced in AY-WB-infected plants on the right compared with non-infected plants on the left. Bar graphs show the percentages of GUS-stained areas of leaves, indicated by red dots. Data are mean ± SD; ^∗^p < 0.05, two-tailed Student’s t-tests.

**Figure S2:**
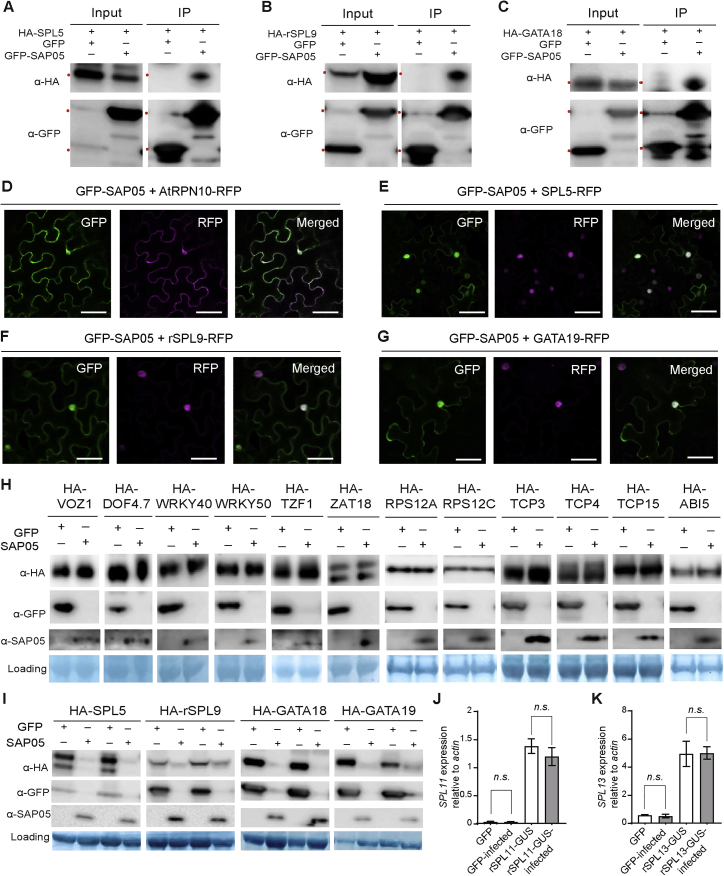
Evidence for the SAP05-mediated target degradation *in planta*, related to Figure 2 (A-C) GFP-SAP05 pulls down HA-tagged SPL and GATA transcription factors from *Nicotiana benthamiana* leaves. Blots labeled as ‘inputs’ show the expression levels of various proteins in *Agrobacterium*-infiltrated *Nicotiana benthamiana* leaves. Blots labeled ‘IP’ show that the HA-tagged TFs co-immunoprecipitate (co-IP) with GFP-SAP05 from the *N. benthamiana* leaves. Co-IPs with GFP alone were included as negative controls. Red dots at left of the blots indicate the expected sizes of TFs. rSPL9: miR156-resistant forms of SPL9. (D-G) Confocal images of GFP-tagged SAP05 with RFP-tagged interacting proteins transiently expressed in *N. benthamiana* leaves. Scale bars, 50 μm. (H) Plant proteins, including several zinc finger transcription factors, that are not reduced in abundance in the presence of SAP05. Western blots of protein extracts from wild-type *A. thaliana* protoplasts transformed with constructs that produce GFP or GFP-SAP05 and HA-tagged VOZ1 (AT1G28520), DOF4.7 (AT4G38000), WRKY40 (AT1G80840), WRKY50 (AT5G26170), TZF1 (AT2G25900) and ZAT18 (AT3G53600) that are all zinc finger transcription factors, HA-tagged RPS12A (AT1G15930) and RPS12C (AT2G32060) that were identified as potential SAP05 interactors in the Hybrigenics Y2H screen and HA-tagged TCP transcription factors TCP3 (AT1G53230), TCP4 (AT3G15030) and TCP15 (AT1G69690) that were identified as potential SAP05 interactors in the *A. thaliana* TF library Y2H screen. Protein loading was visualized using Amido black staining. (I) SAP05 presence reduces SPL/GATA protein abundance in whole *N. benthamiana* leaves. Protein extracts from *N. benthamiana* leaves that were *Agrobacterium*-infiltrated with constructs producing GFP alone or GFP-SAP05 and HA-tagged TFs were subjected to western blot analysis. (J and K) AY-WB phytoplasma infection does not change SPL genes expression. The relative expression levels of *AtSPL11* (J) and *AtSPL13* (K) genes in healthy and AY-WB phytoplasma-infected control (GFP) or overexpression plants (rSPL11-GUS and rSPL13-GUS). The expression levels were shown as fold changes relative to the β actin gene. *N.s.*, no significant difference, two-tailed unpaired Student’s t tests.

To identify potential SAP05 targets in plants, we conducted a yeast two-hybrid (Y2H) screen against an *A. thaliana* seedling cDNA library. SAP05 interacted with several *A. thaliana* zinc-finger transcription factors (TFs), specifically multiple GATA and SPL TFs, which are key regulators of developmental transitions in plants (Figures 2A–2C; Table S1; Ranftl et al., 2016; Xu et al., 2016). We then screened SAP05 against a TF library representing over 78.5% of described *A. thaliana* TFs (Pruneda-Paz et al., 2014). Of 1,957 TFs, 22 genes were identified as potential SAP05 interactors (Table S2), and these included 6 SPLs and 7 GATAs. Further Y2H assays focused on these families revealed a total of 26 GATAs and 12 SPLs from *A. thaliana* that interact with SAP05 (Table S3). GFP-SAP05 also pulled down SPL5, SPL9, and GATA18 from *Nicotiana benthamiana* leaves (Figures S2A–S2C), indicating that SAP05 interacts with SPL and GATA TFs *in planta*. Further, the zinc-finger (ZnF) domains of SPLs and GATAs are sufficient to mediate SAP05 binding in Y2H experiments (Figure 2C).

We also identified the *A. thaliana* 26S proteasome subunit RPN10 as a potential SAP05 interactor in the Y2H screen (Figure 2D; Table S1). RPN10 locates within the 19S regulatory particle of the 26S proteasome and serves as one of the main ubiquitin receptors recruiting ubiquitinated proteins for proteasomal degradation (Fu et al., 1998; van Nocker et al., 1996). RPN10 is composed of two main domains: A N-terminal vWA (von Willebrand factor type A) domain required for proteasome association and a C-terminal half with ubiquitin-interacting motifs (UIMs) involved in recruiting ubiquitinated substrates. We found that SAP05 interacts with the vWA domain but not the UIM domain of AtRPN10 (Figure 2D).

Given that SAP05 interacts with the 26S proteasome subunit RPN10, we investigated whether SAP05 degrades GATA and SPL TFs in plant cells. GFP-SAP05 and AtRPN10-RFP (red fluorescent protein) were detected in the cytoplasm and nuclei and RFP-tagged SPL or GATA proteins in nuclei of plant cells (Figures S2D–S2G), indicating that SAP05, SPL, GATA, and RPN10 locate to the same subcellular locations of plant cells and may interact with each other. Upon transient co-expression of *GATA* or *SPL* genes and *SAP05* (or *GFP* as a control) under control of the constitutive *35S* promoter in *A. thaliana* protoplasts, GATA proteins and SPL proteins were absent or less abundant in the presence of SAP05 compared with GFP (Figure 2E). In contrast, the presence of SAP05 did not reduce the abundance of many other proteins, including 6 randomly selected ZnF TFs, two ribosomal proteins that were identified as potential SAP05 interactors in the *A. thaliana* seedling Y2H screen, three TCP TFs that were identified in the *A. thaliana* TF library Y2H screen, and the TF ABI5, which is degraded by the ubiquitin-proteasome system (UPS) via interaction with RPN10 (Smalle et al., 2003; Figure S2H). Using transient expression assays, we also found SAP05-mediated decrease of GATA and SPL protein levels in whole *Nicotiana benthamiana* leaves (Figure S2I). These data show that SAP05 mediates destabilization of SPL and GATA TFs in plant cells.

**Figure S3:**
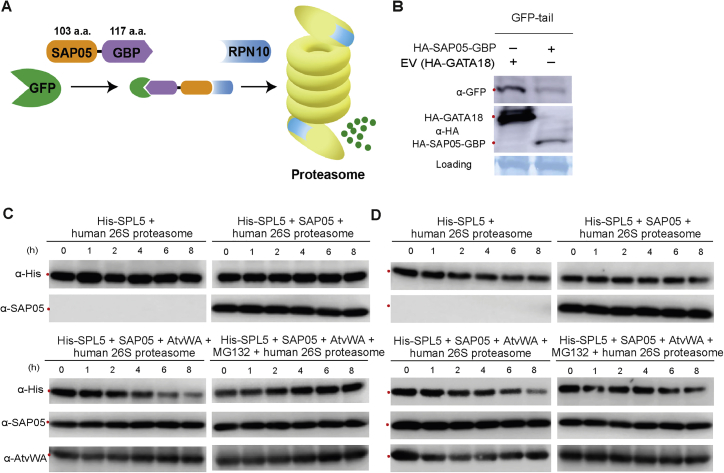
SAP05 can mediate substrate degradation in different systems, related to Figure 3 (A and B) A SAP05-GBP fusion protein mediates the destabilization of GFP in *A. thaliana* protoplast. (A) Proposed model for SAP05-GBP-mediated GFP degradation. (B) western blot analysis of GFP abundance in the presence of SAP05-GBP or an empty vector control when transiently expressed in *A. thaliana* protoplasts. The GBP (GFP-binding protein) derived from a single-chain antibody domain specifically recognizing GFP was fused to SAP05 at its C-ter via a Glycine-rich linker. ‘tail’ represents an unstructured region that serves as an initiation site for proteasomal degradation. (C and D) Purified human 26S proteasomes degrade His-SPL5 in the presence of SAP05 and *A. thaliana* vWA. (C) Repeat 1; (D) Repeat 2. Western blots shown are from protein extracts of recombinant human 26S proteasome preparations (BostonBiochem) in the presence of purified His-SPL5 and SAP05 with or without *A. thaliana* RPN10 vWA (AtvWA) or proteasome inhibitor MG132 probed with antibodies to HA, GFP and SAP05 as shown at left. Red dots at left of the blots indicate the expected sizes of TFs. Protein loading was visualized using Amido black staining.

To investigate the role of AtRPN10 in SAP05-mediated destabilization of plant TFs, we made use of the existing and well-described *A. thaliana* loss-of-function *rpn10* mutant line *rpn10-2* (Lin et al., 2011; Smalle et al., 2003). In protoplasts generated from *rpn10-2* plants, SAP05 no longer degraded GATA18/HAN or SPL5, but when *AtRPN10* was reintroduced into the protoplasts, these TFs were degraded in the presence of SAP05 (Figure 2F). Therefore, AtRPN10 is required for the SAP05-mediated degradation of plant targets.

To investigate whether SPLs are degraded during phytoplasma infection, we made use of lines that express miR156-resistant forms of *SPL11* and *SPL13* (r*SPL11* and r*SPl13*, respectively) fused translationally fused with a β-glucuronidase protein (GUS) under control of their native promoters (Xu et al., 2016). In line with previous findings, *rSPL11::GUS* and *rSPL13::GUS* were expressed in young leaves but not in fully expanded leaves. Newly emerged and developing leaves of phytoplasma-infected plants had visibly reduced GUS staining compared with those of uninfected plants (Figures 2G and 2H), whereas the expression levels of these two genes in those leaves did not differ between healthy and diseased plants (Figures S2J and S2K). These data further support the theory that SPL proteins are destabilized during phytoplasma infection.

### SAP05 bridges host TFs to RPN10 for degradation in the 26S proteasome

**Figure 3:**
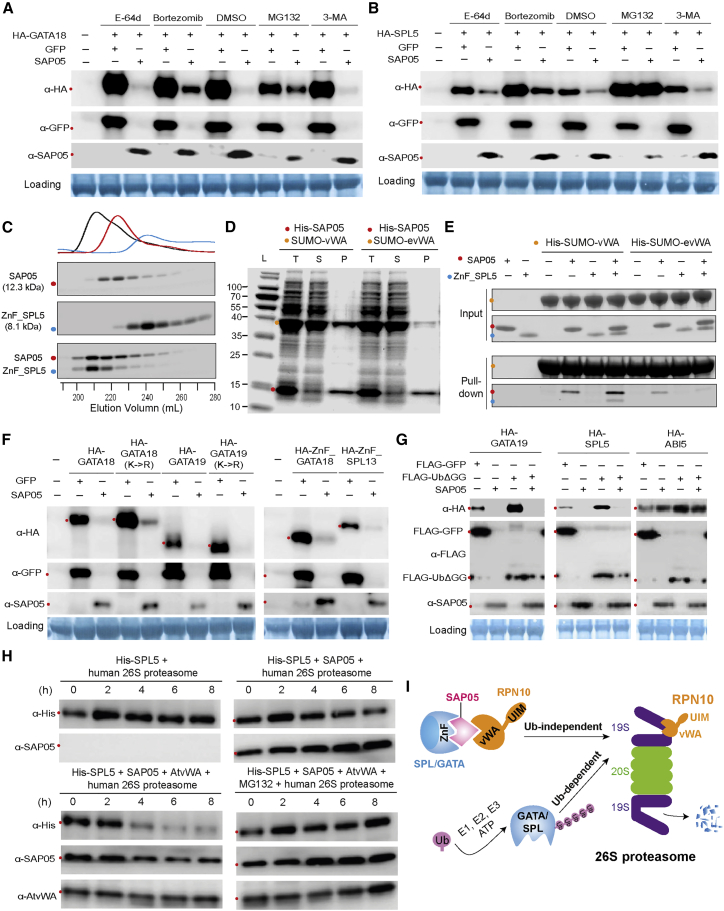
SAP05 hijacks the host ubiquitin receptor RPN10 to destabilize plant GATA and SPL TFs in the 26S proteasome (A and B) 26S proteasome inhibitors reduce SAP05-mediated degradation of plant GATA and SPL in *Arabidopsis* protoplasts. MG132 and bortezomib are 26S proteasome inhibitors. 3-Methyladenine (3-MA) and E-64d are autophagy inhibitors. (C) Direct interaction of SAP05 and the ZnF domain of AtSPL5 in gel filtration assays. Coomassie-stained SDS-PAGE gels with eluted fractions from gel filtration columns are shown. The top graph displays protein elution profiles (UV absorbance at 280 nm) from the column over time. Colored dots at the left of the gels indicate the expected sizes of recombinant proteins in the gels. (D) IMAC co-purification of His-tagged SAP05 and the vWA domain of AtRPN10. evWA, vWA domain mutant with reduced affinity to SAP05 in Y2H assays (Figure 5B); L, ladder; T, total cell extract; S, soluble fraction; P, purified protein. (E) His-tag pull-down of ternary complexes of AtvWA, SAP05, and the AtSPL5 ZnF domain. The His-SUMO-tagged vWA domain or evWA domain were used as bait in IMAC to pull down untagged SAP05 and/or the ZnF domain of AtSPL5. (F) SAP05-mediated degradation does not require lysines in targeted proteins and only requires the SAP05-binding ZnF domain on targets. K > R, all lysines replaced by arginines. (G). GATA19 and SPL5 are degraded in the presence of SAP05 and the dominant-negative ubiquitin variant UbΔGG (an ubiquitin variant lacking the C-terminal double glycine). ABI5, which does not interact with SAP05, is included as a control. (H) Purified human 26S proteasomes degrade His-SPL5 in the presence of SAP05 and *A. thaliana* vWA, and MG132 inhibits this degradation. Repeats of this experiment are shown in Figure S3. (I) A schematic overview of the SAP05-mediated degradation mechanism. SAP05 bridges GATA/SPL TFs to the ubiquitin receptor RPN10 for initiating ubiquitin-independent degradation in the 26S proteasome.

Next we investigated how SAP05 interaction with AtRPN10 leads to degradation of SPLs and GATAs. AtRPN10 is known to be involved in the UPS and autophagy, the two major pathways for protein degradation in eukaryotic cells (Ji and Kwon, 2017; Marshall et al., 2015). Therefore, we wished to investigate the effect of UPS and autophagy in SAP05-mediated degradation of targets. Addition of MG132 and bortezomib, two potent proteasome inhibitors, inhibited SAP05-mediated destabilization of representative GATA and SPL proteins tested (Figures 3A and 3B). In contrast, two autophagy inhibitors, 3-methyladenine and E-64d, did not interfere with SAP05-mediated degradation (Figures 3A and 3B). These results suggest that GATA and SPL proteins are targeted for destabilization by SAP05 in the host 26S proteasome.

SAP05 also mediates degradation of SPL or GATA ZnF domains alone (Figure 3F, right panel), suggesting that direct interactions of SAP05 with these domains are required for substrate degradation initiation. Indeed, SAP05 fused to a GFP nanobody (Rothbauer et al., 2006), a single-chain antibody domain that specifically recognizes GFP, also degraded GFP in *A. thaliana* protoplasts (Figures S3A and S3B). To further investigate whether SAP05 directly binds its plant targets, recombinant proteins or specific domains were expressed in *Escherichia coli* for detecting protein-protein interactions. First, the interaction between SAP05 and the ZnF domain of AtSPL5 was confirmed in gel filtration assays. The two proteins co-eluted in gel filtration with an elution profile distinct from those of the two proteins alone (Figure 3C), suggesting stable *in vitro* complex formation. Moreover, a SUMO-tagged vWA domain was co-purified with His-tagged SAP05 from *E. coli* using immobilized metal affinity chromatography (IMAC) for affinity purification of His-tagged fusion proteins (Figure 3D). In contrast, a mutated vWA domain (38GA39 > HS [evWA]) that does not interact with SAP05 in Y2H assays (further discussed below; Figure 5B), was less enriched during co-purification (Figure 3D). Therefore, SAP05 also binds to the vWA domain *in vitro*. Finally, the His-SUMO-tagged vWA domain pulled down the ZnF domain of AtSPL5 in the presence of SAP05 but not in the absence of this effector, and the evWA domain pulled down less SAP05 and did not pull down the ZnF domain in the presence of SAP05 (Figure 3E). Therefore, SAP05 forms a bridge between the AtSPL5 ZnF and AtRPN10 vWA to generate a ternary complex.

Given that RPN10 functions as one of the main ubiquitin receptors by recruiting poly-ubiquitinated proteins for proteasomal degradation (Fu et al., 1998; Lin et al., 2011), we investigated whether ubiquitination of lysine residues within SAP05 targets is necessary for their degradation. AtGATA18 and AtGATA19 proteins in which all lysines were replaced by arginines were more abundant than wild-type proteins in transient expression assays (Figure 3F, left panel), in agreement with GATAs being short-lived proteins subjected constitutively to degradation by the UPS (Behringer et al., 2014). Nonetheless, both GATA mutants were degraded in the presence of SAP05 (Figure 3F, left panel). Therefore, lysine ubiquitination of substrates may not be required for SAP05-mediated degradation. In support of this idea, co-expression of the dominant-negative ubiquitin Ub-ΔGG, which prevents the ubiquitin chain from conjugating to other proteins (AYu et al., 1997), increased GATA19 and SPL5 protein levels, but both proteins were still degraded in the presence of SAP05 (Figure 3G). In contrast, the protein level of ABI5, which does not interact with SAP05 but is subjected to RPN10-dependent degradation, was increased significantly by UbΔGG overexpression and did not reduce in the presence of SAP05 (Figure 3G). These data indicate that SAP05-mediated target degradation occurs independent of substrate ubiquitination. To directly test this hypothesis, we performed an *in vitro* degradation assay with purified human 26S proteasomes. Addition of SAP05 and His-SPL5 did not result in degradation of the latter (Figure 3H, top right panel). However, when the purified AtRPN10 vWA domain was also added to the assay, His-SPL5 was degraded in the presence of SAP05 (Figure 3H, bottom left panel). Moreover, MG132 inhibited His-SPL5 degradation in the presence of SAP05 and AtRPN10 vWA (Figure 3H, bottom right panel). These results suggest that SAP05 and the *A. thaliana* vWA domain are required for His-SPL5 degradation in the human 26S proteasome. These data are in agreement with our finding that SAP05 links AtSPL5 and AtRPN10 vWA to form a ternary complex (Figure 3E). Because the purified recombinant human 26S proteasomes lack ubiquitin conjugation enzymes, SAP05 apparently initiates degradation of SAP05 interactors in the 26S proteasome without the need for substrate ubiquitination. These results indicate that SAP05 mediates SPL/GATA degradation in a ubiquitination-independent manner by hijacking the plant host 26S proteasome component RPN10 (Figure 3I).

### Concurrent destabilization of SPLs and GATAs by SAP05 effectors decouples plant developmental transitions

**Figure 4:**
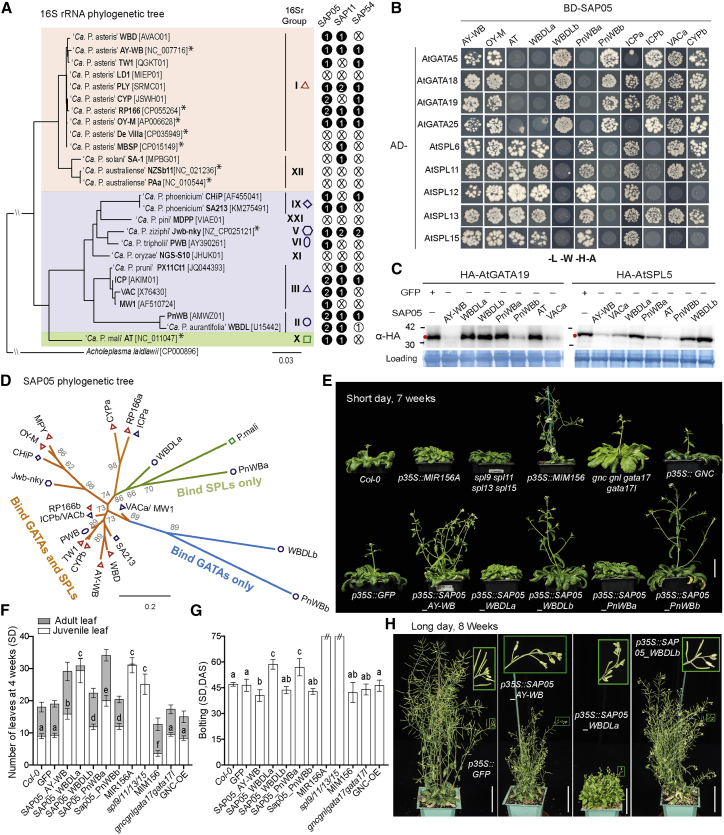
Phytoplasma SAP05 family effectors differentially bind and degrade plant SPL and GATA TFs (A) SAP05 homologs are present in divergent phytoplasmas. The phylogenetic tree is based on phytoplasma 16S rRNA gene alignment (Chung et al., 2013). The three distinct clades (indicated in pink, purple, and green) and 16Sr groups are shown. The black circles indicate the numbers of full-length SAP05, SAP11, and SAP54 effector genes found in each phytoplasma. For white circles, numbers indicate partial genes and crosses indicate absence. ^∗^, phytoplasmas for which genomes were sequenced to completion. (B) Interactions of SAP05 homologs from divergent phytoplasmas and representative *A. thaliana* (At) GATA and SPL proteins in Y2H assays. See the legend of Figure 2 for abbreviations. Yeast growth on -L-W medium is shown in Figure S6B. (C) SAP05 homologs of divergent phytoplasmas degrade At GATA19 and SPL5 in At protoplasts. See the legend of Figure 2 for abbreviations. (D) SAP05 phylogenetic tree based on alignment of SAP05 amino acid sequences. Phytoplasma names and symbols to indicate 16Sr groups are shown in Figure 4A. Branch lengths correspond to the number of amino acid changes. Bootstrap values are indicated at the nodes. (E) Morphologies of wild-type plants, *GFP*-expressing control plants, plants expressing different SAP05 homologs, and transgenic plants with altered expression of *MIR156/SPL* or *GATA* genes. Scale bar, 4 cm. (F and G) Statistical analysis of phenotypes shown in (E): numbers of rosette leaves of 4-week-old plants (F) and time of shoot emergence from rosettes (bolting time; G). // indicates that the plants did not bolt at the time of observation. Data are mean ± SD; different letters indicate significant difference based on multiple comparisons (Turkey method) after ANOVA. (H) Morphologies of GFP control plants and plants expressing different SAP05 homologs grown under LD conditions. Insets show enlarged images of inflorescence on mature plants. Scale bar, 4 cm.

We identified one or two *SAP05* homologs in 17 phytoplasmas from 7 of 10 16S rDNA (16Sr) clades (Figure 4A). Most of these homologs interacted with SPLs and GATAs (Figure 4B). However, for witches’ broom disease of lime (WBDL) phytoplasma and peanut witches’ broom (PnWB) phytoplasmas, which have two SAP05 genes, one SAP05 interacted only with SPLs and the other only with GATAs. In contrast, SAP05 of *Ca*. Phytoplasma *mali* (AT) only interacted with SPLs. Consistent with these binding specificities in yeast, the SAP05 homologs degraded one or both representative members of the SPL and GATA families in protoplast destabilization assays (Figure 4C). Phylogenetic analyses of the SAP05 effectors revealed distinct subclades of SAP05 homologs that bind and degrade both TFs or only SPLs or GATAs (Figure 4D). These data indicate that some SAP05 homologs have evolved to differentially interact and degrade plant SPL and GATA TFs.

**Figure S4:**
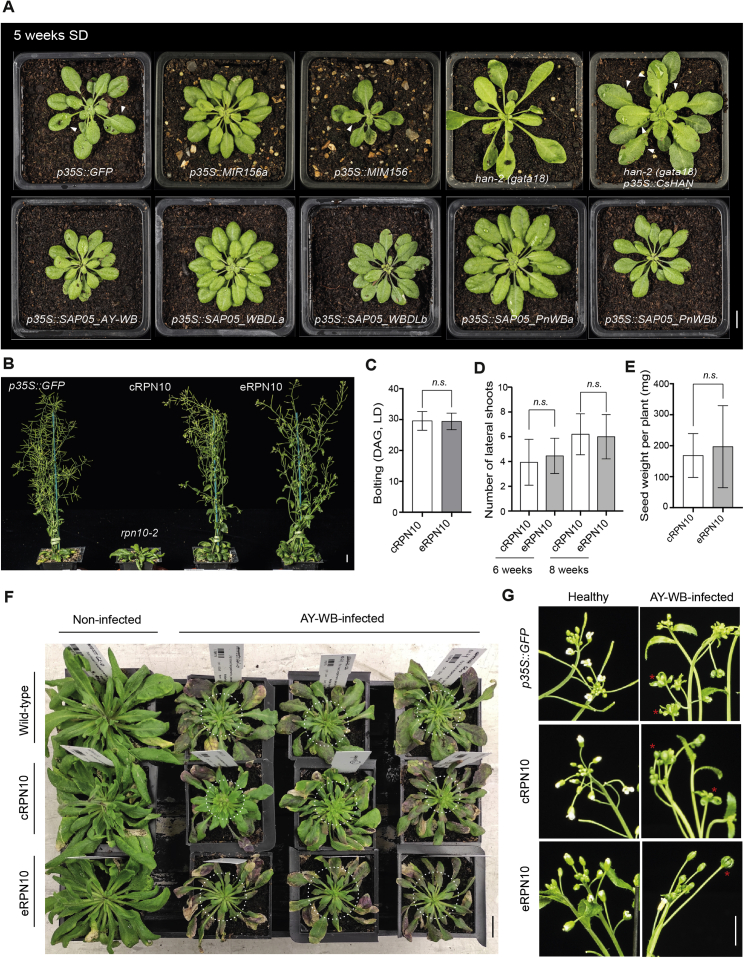
Phenotypic analysis of transgenic plants or plants infected with AY-WB phytoplasma, related to Figures 4 and 5 (A) Different SAP05 homologs induce distinct leaf morphologies in *A. thaliana* reminiscent of either *MIR156* overexpression or a *GATA* mutant. Arrowheads indicate leaf serrations. The *A. thaliana han* mutant (*han-2*) produces rosettes with a smooth margin while the overexpression of a *Cucumis sativus L. GATA18* homolog (*CsHAN*) under the control of the 35S promoter in the *A. thaliana han-2* background leads to leaves with more severe serrations. Scale bar, 1 cm. (B) An engineered *RPN10* allele rescues the developmental defects of the *rpn10-2* mutant. The *rpn10-2* mutant was complemented by either a wild-type *AtRPN10* allele (cRPN10) or an engineered RPN10 allele (AtRPN10 m1, eRPN10) under the control of the native promoter. At least two independent lines for each complementation were obtained, with consistent plant phenotype. Scale bar, 1 cm. (C-E) Statistical analysis of the flowering time (C), branching (D) and seed weight (E) of cRPN10 plants and eRPN10 plants. The number of lateral shoots was scored both at 6 weeks after germination and 8 weeks after germination. Data are mean ± SD; *n.s.*, no significant difference, two-tailed unpaired Student’s t tests. (F) Rosette leaf morphology on healthy and AY-WB phytoplasma-infected plants. All plants were kept in short-day conditions throughout the experiment. Circled areas correspond to rosette leaves that emerged during phytoplasma infection. Scale bar, 1 cm. (G) eRPN10 plants show phyllody symptoms during AY-WB infection. Typical flower morphology on healthy plants or infected plants is shown. Asterisks indicate leaf-like flowers on infected plants. Scale bar, 1 cm.

SPLs regulate plant developmental phase transitions, and most are regulated developmentally by microRNA156 (miR156) (Xu et al., 2016), whereas GATA proteins regulate photosynthetic processes, leaf development, and flower organ development (Ding et al., 2015; Ranftl et al., 2016; Richter et al., 2010). Stable transgenic *A. thaliana* plants that constitutively express genes of the SPL-interacting SAP05_WBLDa_ or SAP05_PnWBa_ phenotypically resembled *miR156* overexpression plants or a high-order *spl* mutant (*spl9 spl11 spl13 spl15*) (Figures 4E–4H), including production of more juvenile leaves (Figure 4F) and delayed flowering (Figure 4G), unlike *MIM156* plants, in which *miR156* activity is reduced via target mimicry (Franco-Zorrilla et al., 2007). In contrast, transgenic plants expressing GATA-interacting SAP05_WBLDb_ or SAP05_PnWBb_ did not show much difference in production of juvenile leaves compared with wild-type plants. A quadruple *gata* mutant, *gnc gnl gata17 gata17l*, or the overexpression of a GATA member, *GNC*, which significantly impair plant greening and growth (Ranftl et al., 2016; Richter et al., 2010), did not alter the juvenile leaf number. However, phenotypes of SAP05_WBLDb_ and SAP05_PnWBb_ plants resembled those of well-characterized *gata* mutants regarding early flowering (Figures 4E and 4G; Ranftl et al., 2016; Richter et al., 2010, 2013), more secondary branches combined with reduced height (Figure 4H; Ranftl et al., 2016), and narrower rosette leaves with smooth margins (Figure S4A; Ding et al., 2015). Noticeably, SAP05 homologs that bind either SPLs or GATAs did not induce the full witches’ broom-like phenotypes that were observed in plants expressing the SAP05 homologs that bind both SPL and GATA TFs. This suggests that the concurrent destabilization of SPLs and GATAs by SAP05 decouples normal plant juvenile-to-adult and vegetative-to-reproductive developmental transitions, generating plants that retain juvenile characteristics and nonetheless bolt to produce flowering shoots that remain sterile. Therefore, the combination of SAP05-mediated SPL and GATA degradation leads to induction of the witches’ broom phenotype.

**Figure S5:**
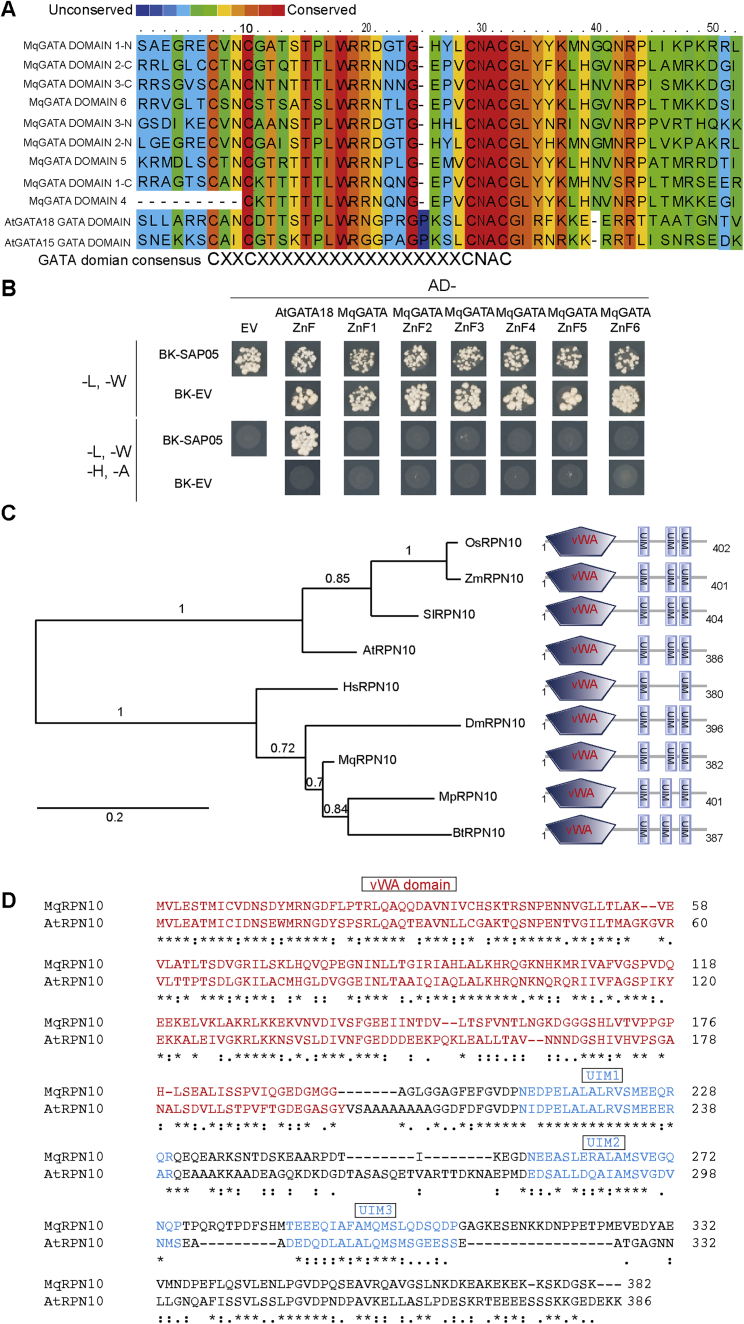
Sequence alignment of plant and metazoan TF GATA domains and RPN10 proteins, related to Figure 5 (A) The alignment of *M. quadrilineatus* GATA domains with two *A. thaliana* GATA domains. (B) Y2H analysis of SAP05 interaction with various *M. quadrilineatus* GATA domains. The GATA domain of AtGATA18 was used as a positive control. (C) Phylogenetic analysis of RPN10 proteins from various organisms. The presence of vWA and UIM domains were predicted by PFAM. AtRPN10, *Arabidopsis thaliana* RPN10 (Uniprot ID: P55034); SlRPN10, *Solanum lycopersicum* RPN10 (Uniprot ID: A0A3Q7F6N7); OsRPN10, *Oryza sativa* RPN10 (Uniprot ID: O82143); ZmRPN10, *Zea mays* RPN10 (Uniprot ID: B6TK61); DmRPN10, *Drosophila melanogaster* RPN10 (Uniprot ID: P55035); HsRPN10, *Homo sapiens* RPN10 (Uniprot ID: Q5VWC4); BtRPN10, *Bemisia tabaci* RPN10 (GenBank: XP_018915695); MqRPN10, *Macrosteles quadrilineatus* RPN10; MpRPN10, *Myzus persicae* RPN10 (GenBank: XP_022181722.1). (D) Sequence alignment of the *A. thaliana* RPN10 and the *M. quadrilineatus* RPN10 proteins. The vWA domains and UIM domains are highlighted in red and blue, respectively.

**Figure S6:**
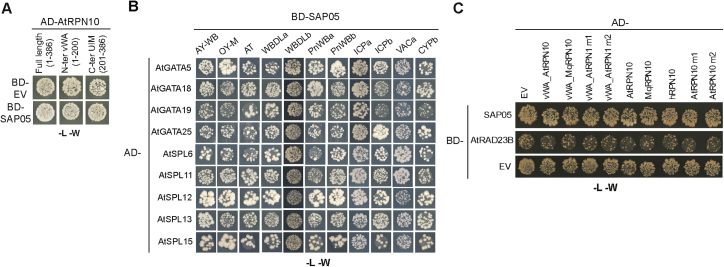
Yeast transformation controls for Y2H assays in the study, related to Figures 2, 4, and 5 (A-C) Yeast growth on medium lacking leucine and tryptophan, indicating presence of the AD and BD constructs in yeast two-hybrid assays for Figures 2D (A), 4B (B) and 5B (C) in the main text.

### Engineering plant RPN10 for resistance to SAP05 activity

Animals encode GATA TFs and RPN10 proteins but lack SPLs. To investigate whether SAP05 also interacts with GATA proteins of the leafhopper vector, we mined the transcriptome assembly of *M. quadrilineatus* (Drurey et al., 2019) for GATA TFs and identified six distinct transcripts with typical GATA ZnF domains (Figure S5A). None of these insect ZnF domains interacted with SAP05 in Y2H assays (Figure S5B).

**Figure 5:**
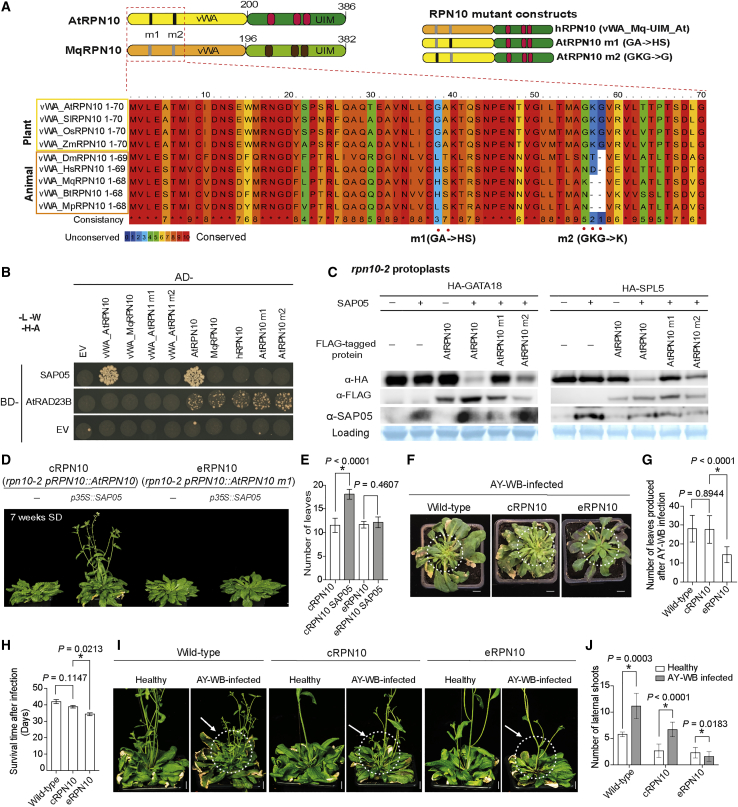
Insect-directed engineering of At RPN10 confers resistance to SAP05 action (A) Schematic of domain organizations of At and *M. quadrilineatus* RPN10 proteins and alignment of the first 70 residues of the vWA domains. Highly divergent residues are highlighted below the alignment. Alignments of full-length RPN10 homologs are shown in Figure S5D. (B) Specific residues within the At RPN10 vWA domain are required for SAP05 interaction in Y2H assays. See the legends of Figures 2 and 5A for abbreviations. Yeast growth on -L-W medium is shown in Figure S6C. (C) Specific residues within the At RPN10 vWA domain are required for SAP05 degradation of plant GATA and SPL in At protoplasts. See the legend of Figure 2E. (D–J) Specific residues within the At RPN10 vWA domain are required for leaf and stem proliferation of At plants in the presence of constitutively expressed SAP05 (D and E) and during AY-WB phytoplasma infection (F–J). At plants included in these experiments were *rpn10-2* null mutants complemented with wild-type AtRPN10 (cRPN10) or AtRPN10 m1 (eRPN10). Scale bars, 1 cm. Symptomatic leaves in (F) and lateral shoots in (I) are circled. Phenotypes were analyzed statistically for number of leaves of 4-week-old plants (E), number of newly produced rosette leaves after AY-WB infection (G), plant survival time after AY-WB inoculation (H), and numbers of lateral shoots in control and infected plants (J). Data are mean ± SD from 2 independent experiments. ^∗^p < 0.05, two-tailed Student’s t test.

We also identified a *M. quadrilineatus* RPN10 homolog (MqRPN10) that is highly similar in sequence to *A. thaliana* RPN10 (AtRPN10) (Figure 5A; Figures S5C and S5D). SAP05 of AY-WB did not interact with MqRPN10 in Y2H assays, nor with the MqRPN10 vWA domain or a hybrid RPN10 (hRPN10) consisting of the *M. quadrilineatus* vWA domain and the *A. thaliana* C-terminal (UIM) domain (Figure 5B). Comparison of multiple vWA domains of plant and animal RPN10 homologs revealed differences between the two groups in two regions corresponding to amino acids 38–39 (GA versus HS) and 56–58 (GKG versus K–) in the *A. thaliana* vWA domain (Figure 5A). Altering these residues within *A. thaliana* RPN10 to those present in the *M. quadrilineatus* homolog to create RPN10_38GA39 > HS (m1) and RPN10_56GKG58 > K (m2) resulted in loss of SAP05 binding in Y2H assays (Figure 5B). The RPN10 variants interacted with the *A. thaliana* RADIATION SENSITIVE23 (RAD23B) protein, a ubiquitin shuttle factor that binds RPN10 UIM domains (Farmer et al., 2010), indicating that the RPN10 variants are functional in Y2H assays. In addition, SAP05 degradation assays of AtGATA18 and AtSPL5 in *A. thaliana rpn10-2* protoplasts showed that these SAP05 targets were less degraded in the presence of AtRPN10 m1 compared with AtRPN10 or AtRPN10 m2 (Figure 5C), indicating that the 38GA39 residues mediate SAP05 binding and activities. This result is in agreement with our finding that SAP05-mediated degradation of His-SPL5 by the human 26S proteasome occurred only in the presence of the AtRPN10 vWA domain (Figure 3H) because SAP05 is unlikely to interact with PSMD4, the human RPN10 homolog, because of the 38HS39 residues in its vWA domain. In addition, plant SUMO-tagged RPN10 vWA domains that carried the 38GA39 > HS mutations (SUMO-evWA) had lower affinity for SAP05 in *E. coli* lysates compared with the wild-type RPN10 vWA domain (Figure 3D), and the His-SUMO-tagged evWA domain did not pull down ZnF domain of AtSPL5 in the presence of SAP05 but His-SUMO-tagged vWA did (Figure 3E). Therefore, 38GA39 mediate direct binding of SAP05 of AtRPN10 *in vitro* and *in vivo.*

Next, we considered the possibility of engineering plant RPN10 as a way to block SAP05 activities. Even though *rpn10* null mutants have severe growth defects (Lin et al., 2011; Smalle et al., 2003), the SAP05 non-interacting allele *AtRPN10 m1* (called eRPN10 for engineered RPN10) largely rescued the developmental defects of the *rpn10-2* plants and were similar in habit to cRPN10 plants (complementation RPN10 carrying wild-type *AtRPN10*) (Figures S4B–S4E). Transformation of the *p35S::SAP05* construct in the eRPN10 background generated wild-type-looking plants without obvious developmental phenotypes in contrast to that in cRPN10 plants (Figures 5D and 5E). Therefore, the RPN10_38GA39 > HS mutation confers resistance to phytoplasma-SAP05-mediated developmental changes in *A. thaliana*.

To investigate the contribution of SAP05 to symptom development because of AY-WB phytoplasma infection in *A. thaliana*, we infected wild-type, cRPN10, and eRPN10 plants with AY-WB phytoplasma. The infected wild-type and cRPN10 plants produced more small, deformed leaves and more lateral shoots compared with plants of similar age not infected with phytoplasma (Figures 5F–5J; Figure S4F). The symptoms of these infected plants resembled the phenotypes of *p35S::SAP05* (Figures 1A–1C) and cRPN10 *p35S::SAP05* plants (Figure 5D). In contrast, eRPN10 plants infected with phytoplasma did not produce severely deformed leaves nor an increased number of lateral shoots compared with non-infected plants (Figures 5F–5J; Figure S4F). Moreover, the leaves of infected eRPN10 *A. thaliana* plants showed enhanced reddening compared with wild-type or cRPN10 plants (Figure 5F; Figure S4F), indicating that SAP05 actions may reduce plant stress-induced senescence during phytoplasma infection. Indeed, AY-WB-infected eRPN10 plants died earlier compared with cRPN10 and wild-type plants (Figure 5H). All AY-WB-infected *A. thaliana* genotypes produced leaf-like flowers (Figure S4G) that resemble the phyllody symptoms of AY-WB-infected plants, indicating that the engineered RPN10 allele does not interfere with the leaf-like flower phenotype induced by another AY-WB phytoplasma effector, SAP54. These data demonstrate that the AY-WB SAP05 effector is largely responsible for the shoot proliferation/witches’-broom-like symptoms during AY-WB infection of *A. thaliana* and that blocking SAP05 activities reduces host tolerance toward this phloem-inhabiting, insect-vectored bacterial pathogen.

## Discussion

**Figure 6:**
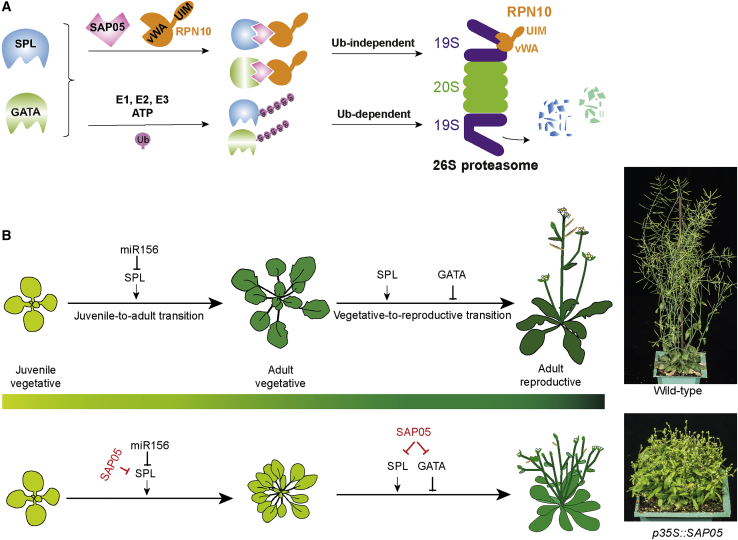
Model of SAP05-induced delayed aging and witches’ broom formation (A) A schematic of SAP05-mediated selective protein degradation in the plant host 26S proteasome. (B) During normal plant growth, *SPL* genes are regulated by *miRNA156* to ensure proper progression of plant developmental phase transitions, whereas *GATA* genes regulate multiple processes of plant development, including suppression of flowering and branching (top panel). In phytoplasma-infected plants, the phytoplasma effector SAP05 degrades SPL and GATA transcription factors (TFs). SPLs are positive regulators of the juvenile-adult and adult-reproductive transitions, and GATA TFs are inhibitors of the formation of flowering branches. Consistent with the functions of these TFs, the actions of SAP05 lead to the induction of juvenilized plants that produce flowering branches. This uncoupling of plant developmental phase transitions by phytoplasma SAP05 contributes to the witches’ brooms formation.

This work shows that SAP05 effector co-opts the plant 26S proteasome ubiquitin receptor RPN10 to mediate degradation of SPL and GATA—two distinct classes of plant TFs—through a ubiquitination-independent process (Figure 6A). SPL TFs have a conserved role in controlling developmental phase transitions of vascular plants (Huijser and Schmid, 2011; Wang et al., 2009; Xu et al., 2016), whereas GATA TFs regulate plant organ development, timing of flowering, and branching patterns in dicots and monocots (Ding et al., 2015; Hudson et al., 2013; Ranftl et al., 2016; Richter et al., 2010; Zhang et al., 2013). SAP05 binding and the degradation spectrum against SPLs or GATAs and the phenotypes of plants expressing SAP05 homologs are consistent with known functions of SPLs and GATAs (Figures 4D–4H). For example, SAP05, which simultaneously degrades SPLs and GATAs, decouples plant developmental phase transitions and causes delayed plant aging, witches’ broom-like excessive vegetative tissue, and sterile adult shoot production (Figures 4D–4H and 6B). Our model describes a mechanistic framework for how obligate parasites can induce complex and substantial phenotypic changes in their hosts in ways that favor their transmission to other trophic levels.

SAP05-induced decoupling of host developmental transitions likely promotes plant developmental traits that facilitate survival and spread of this “zombie plant” pathogen. Phytoplasmas are restricted to the phloem. These bacteria may benefit from SAP05-induced phenotypes by increasing the numbers of leaves and adult shoots to generate more vascular tissue the phytoplasmas can colonize. In support of this notion, engineered RPN10 plants that are resistant to SAP05 activities do not produce more vegetative tissue and die earlier during phytoplasma infection (Figures 5F–5J). Moreover, although flower structures are formed, they remain sterile. So photosynthates transported within the phloem are not being used for flowering and seed production and may instead be consumed by the phytoplasmas, which are known to accumulate in plant sink tissues. Enrichment of nutrients is likely important because phytoplasmas lack essential metabolic pathways and depend on import of a diverse range of metabolites, including sugars, nucleotides, amino acids, and ions, mostly via ABC transporters (Bai et al., 2006; Oshima et al., 2004). Finally, a delay in plant senescence and death increases the likelihood of plants being visited and colonized by phytoplasma insect vectors, promoting the number of insects that can transmit the phytoplasmas. This effect may be intensified by insect vectors being more attracted to symptomatic phytoplasma-infected plants and the activities of SAP11 and SAP54 effectors that enhance insect attraction and colonization (Al-Subhi et al., 2020; MacLean et al., 2014; Orlovskis & Hogenhout, 2016; (Sugio et al., 2011a)).

Our finding that SAP05 does not bind RPN10 of the leafhopper vector led to a strategy for engineering plants to be insensitive to SAP05 activities. The SAP05-binding specificity to RPN10 can be dependent on just two amino acids that, fascinatingly, are one of the only few sequence differences between plant and human/animal RPN10 vWA domains (Figure 5A). eRPN10 plants carrying AtRPN10 GA > HS are more resistant to SAP05 activity during phytoplasma infection. Hence, introduction of single-nucleotide changes in RPN10 genes (for example, by CRISPR-Cas technologies; Chen et al., 2019) is a promising strategy to achieve durable resistance of crops to phytoplasmas.

SAP05 bridges TFs and a conserved proteasome component through a biochemical mechanism that results in target degradation without ubiquitination. Some human proteins, such as FAT10 (HLA-F locus adjacent transcript 10) and its co-factor NUB1L (NEDD8 ultimate buster-1 long) (Rani et al., 2012), are known to bind the vWA domain. Unlike SAP05, FAT10 shares sequence similarity with ubiquitin (Schmidtke et al., 2014). The FAT10/NUB1L pathway also requires E1/E2/E3 ligases and the presence of lysine residues on the substrate targeted for degradation (Rani et al., 2012). Unlike SAP05 (Figure 3H; Figures S3C and S3D), FAT10 is degraded along with its substrate (Hipp et al., 2005.) Therefore, the mode of action of SAP05 is different from that of FAT10/NUB1L despite both binding the RPN10 vWA domain. This distinct mode of action of SAP05 has potential practical applications given that targeted protein degradation (TPD) has emerged as a promising approach for drug discovery (Chamberlain and Hamann, 2019). Phytoplasma SAP05 effectors may be used as alternatives to other TPD systems, such as proteolysis-targeting chimera (PROTAC), which uses small-molecule ligands to create complexes between E3 ligases and targets (Schapira et al., 2019). Phytoplasma SAP05 effectors may enable a more direct TPD technology that is independent of E3 ligases.

In contrast to most pathogens effectors that target immune responses of their hosts, this and previous studies show that phytoplasma effectors have converged onto modulating key plant developmental regulators. Our finding that SAP05 effectors mediate degradation of SPLs and GATAs that control precise developmental stage transitions enables phytoplasmas to take control of their plant host and corroborates the view that the developmental changes enforced by multi-host obligate phytoplasma parasites are adaptive.

## Limitations of the study

This study used the model plant *A. thaliana*. With advancement of CRISPR-mediated gene targeting tools that enable DNA substitutions, it may soon be possible to introduce GA > HS mutations in RPN10 of multiple crops afflicted by phytoplasma infection. Although the phytoplasma-infected *A. thaliana* RPN10_GA > HS_ mutants were more resistant to actions of SAP05, other symptoms, including phyllody induced by the AY-WB SAP54 effector, were still observed.

## STAR★Methods

### Key resources table

**Table:** 

REAGENT or RESOURCE	SOURCE	IDENTIFIER
**Antibodies**		

Anti-SAP05 antibody	this study	RRID:AB_2893243
Anti-HA tag antibody	Eurogentec	Cat# MMS-101R; RRID:AB_291262
Anti-GFP antibody	Santa Cruz Biotechnology	Cat# sc-9996; RRID:AB_627695
Anti-FLAG antibody	Sigma	Cat# F-3165; RRID:AB_259529
Penta·His Antibody	QIAGEN	Cat# 34660; RRID:AB_2619735
Anti-AtRPN10	Agrisera	Cat# AS194266; RRID:AB_2893242

**Bacterial and virus strains**		

Aster yellows witches’-broom phytoplasma	this study	Taxonomy ID: 322098
*Escherichia coli* BL21(DE3)	Novagen	Cat# 70235
*Agrobacterium tumefaciens* GV3101	N/A	N/A

**Chemicals, peptides, and recombinant proteins**		

Human 26S Proteasome	R&D Systems	Cat# E-365-025
MG132	Sigma	Cat# M7449
Bortezomib	Sigma	Cat# 5043140001
E-64d	Sigma	Cat# E8640
3-MA	Sigma	Cat# M9281
GFP-Trap Magnetic Agarose	ChromoTek	Cat# gtma-20

**Experimental models: Organisms/strains**		

Yeast strain AH109	N/A	N/A
Yeast strain NMY51	N/A	N/A
*Arabidopsis thaliana* Col-0	N/A	N/A
*Arabidopsis thaliana* overexpressor line *p35S::GFP*	this study	N/A
*Arabidopsis thaliana* overexpressor line *p35S::SAP05_AY-WB*	this study	N/A
*Arabidopsis thaliana* overexpressor line *pAtSUC2::GFP*	this study	N/A
*Arabidopsis thaliana* overexpressor line *pAtSUC2::SAP05*	this study	N/A
*Arabidopsis thaliana* overexpressor line *pSPL13::rSPL13-GUS*	NASC	NASC ID: N69817
*Arabidopsis thaliana* overexpressor line *pSPL11::rSPL11-GUS*	NASC	NASC ID: N69815
*Arabidopsis thaliana* mutant line *rpn10-2*	NASC	NASC ID: N366730
*Arabidopsis thaliana* overexpressor line *p35S::MIR156A*	NASC	NASC ID: N67849
*Arabidopsis thaliana* mutant line *spl9 spl11 spl13 spl15*	NASC	NASC ID: N69797
*Arabidopsis thaliana* overexpressor line *p35S::MIM156*	NASC	NASC ID: N9953
*Arabidopsis thaliana* mutant line *gnc gnl gata17 gata17l*	Ranftl et al., 2016	N/A
*Arabidopsis thaliana* overexpressor line *p35S::GNC*	Ranftl et al., 2016	N/A
*Arabidopsis thaliana* overexpressor line *p35S::SAP05_WBDLa*	this study	N/A
*Arabidopsis thaliana* overexpressor line *p35S::SAP05_WBDLb*	this study	N/A
*Arabidopsis thaliana* overexpressor line *p35S::SAP05_PnWBa*	this study	N/A
*Arabidopsis thaliana* overexpressor line *p35S::SAP05_PnWBb*	this study	N/A
*Arabidopsis thaliana rpn10-2* mutant wild-type allele complementation line cRPN10	this study	N/A
*Arabidopsis thaliana rpn10-2* mutant engineered m1 allele complementation line *eRPN10*	this study	N/A
*Arabidopsis thaliana* cRPN10 X *p35S::SAP05* line	this study	N/A
*Arabidopsis thaliana* eRPN10 X *p35S::SAP05* line	this study	N/A
*Arabidopsis thaliana* mutant line *han-2*	Ding et al., 2015	N/A
*Arabidopsis thaliana han-2* X *p35S::CsHAN*	Ding et al., 2015	N/A

**Recombinant DNA**		

pB7WG2 (gene specific constructs listed in Table S5)	VIB-UGent Center for Plant Systems Biology	Vector ID: 1_04
pHW59 (gene specific constructs listed in Table S5)	Mathieu et al., 2007	N/A
pEarleyGate202 (gene specific constructs listed in Table S5)	Tair	Stock# CD3-688
pB7WGF2 (gene specific constructs listed in Table S5)	VIB-UGent Center for Plant Systems Biology	Vector ID: 1_42
pB7RWG2 (gene specific constructs listed in Table S5)	VIB-UGent Center for Plant Systems Biology	Vector ID: 1_63
pBI121 (gene specific constructs listed in Table S5)	Tair	Stock# CD3-388
pGADT7 (gene specific constructs listed in Table S5)	Addgene	Plasmid #61702
pGBKT7 (gene specific constructs listed in Table S5)	Addgene	Plasmid #61703
pGADHA (gene specific constructs listed in Table S5)	*Dualsystems Biotech*	N/A
pLEXA-C (gene specific constructs listed in Table S5)	*Dualsystems Biotech*	N/A
pUGW15 (gene specific constructs listed in Table S5)	Pecher et al., 2019	N/A
pOPINF (gene specific constructs listed in Table S5)	Berrow et al., 2007	N/A
pOPINS3C (gene specific constructs listed in Table S5)	Berrow et al., 2007	N/A
pOPINA (gene specific constructs listed in Table S5)	Berrow et al., 2007	N/A

**Software and algorithms**		

Prism 7	Graphpad	https://www.graphpad.com/scientific-software/prism/
Fiji	ImageJ	https://imagej.net/software/fiji/
Phylogeny.fr	(Dereeper et al., 2008)	http://www.phylogeny.fr/index.cgi

### Resource availability

#### Lead contact

Further information and requests for resources and reagents should be directed to and will be fulfilled by the Lead Contact, Saskia A. Hogenhout (saskia.hogenhout@jic.ac.uk).

#### Materials availability

Plasmids, transgenic lines and antibodies generated in this study will be made available on request, but we may require a payment and/or a completed Materials Transfer Agreement if there is potential for commercial application.

### Experimental model and subject details

#### *Arabidopsis* growth

*A. thaliana* Columbia-0 ecotype (*Col-0*) plants were grown in the greenhouse under either long-day (16 h light/8 h dark) or short-day conditions (10 h light/14 h dark) at 22°C. Plant age was determined from the date seeds were transferred to growth chambers after stratification. Juvenile leaves refer to rosette leaves that only produce trichomes on their adaxial side. Rosette leaves that have trichomes on both side of leaves (adaxial and abaxial) were recorded as adult leaves. Bolting time was recorded when the main inflorescence reached a height of 0.5 cm. Transgenic plants were generated as previously described (MacLean et al., 2011). For generating *p35S::SAP05* or *pAtSUC2::SAP05* plants, codon-optimized SAP05 coding sequences (without the secretory signal peptide) were used. SAP05 sequences used for generating transgenic plants were listed in Table S4.

#### AY-WB phytoplasma maintenance

*M. quadrilineatus* colonies carrying the AY-WB phytoplasma (Sugio et al., 2011b) were reared on infected lettuce (*Lactuca sativa*) and China aster (*Callistephus chinensis* Nees) under long-day conditions at 24°C.

### Method details

#### Yeast two-hybrid analysis

The initial Y2H screen of SAP05 against an *A. thaliana* seedling library was performed by Hybrigenics Services SAS (Paris, France). The coding sequence of SAP05 without the secretory signal peptide was cloned into a pB27 bait plasmid as a C-terminal fusion to the LexA domain (Table S1). The prey library was constructed from an *A. thaliana* seedling cDNA library, with pP6 as the prey plasmid. A total 65.2 million clones were screened. In a second yeast two-hybrid screen (Table S2), the same SAP05 sequence was cloned into the pDEST32 plasmid and screened against an *A. thaliana* transcription factor library (pDEST22-TF), as previously described (de Folter and Immink, 2011). The identified interactions were further confirmed using the Matchmaker Gold yeast two-hybrid system (Clontech) or the DUALhybrid system (Dualsystems Biotech). Yeast growth on medium lacking leucine and tryptophan (-L-W) indicates presence of AD and BD constructs and on medium lacking leucine, tryptophan, histidine and alanine (-L-W-H-A) interactions between the AD and BD fusion proteins. Yeast plates were kept in 28°C growth chambers for 5 days before imaging. SPL and GATA proteins identified in these screens are summarized in Table S3.

#### Protoplast degradation assays

*A. thaliana* (Col-0) mesophyll protoplast isolation and transformation were carried out as reported (Yoo et al., 2007). Briefly, mesophyll protoplasts were isolated from leaves of 4–5-week-old *A. thaliana* plants grown under short-day conditions. For transfection, 100 μL of fresh protoplast solution (40,000 protoplasts) was transformed with 8 μg of high-quality plasmids (4 μg each for co-transfection) using the PEG-calcium method. Transfected protoplasts were incubated at room temperature (22-25°C) for 16 h in the dark before harvest. For drug treatment, a final concentration of 20 μM MG132 (Sigma), 5 μM Bortezomib (Sigma), 10 μM E-64d (Sigma) or 5 mM 3-MA (Sigma) were added during the 16-h incubation period. Except for 3-MA which was prepared as 0.1 M stock solution in water, the others were prepared as 10 mM stock solution in DMSO. Equivalent volume of DMSO was used as mock control. For detection of proteins on western blots, whole protein extracts from protoplasts were separated on NuPAGE 4%–12% Bis-Tris Gels (Invitrogen) and transferred to 0.45-μm PVDF membranes (Thermo Scientific) using the Bio-Rad mini-PROTEAN Electrophoresis system. Membranes were blocked by incubation in 5% (w/v) milk power in phosphate-buffered saline and 0.1% (v/v) Tween-20 for 2 h at room temperature. Primary antibody incubation was carried out at 4°C overnight. Antibody to SAP05 from AY-WB phytoplasma were raised to the mature part of the SAP05 protein (residues 33–135), which was produced with a 6XHis-tag into *E. coli* and purified. The purified protein was used for raising polyclonal antibodies in rabbits (Genscript). Optimal detection of SAP05 in phytoplasma-infected plants occurred at a 1:2,000 dilution of the antibody, and this dilution was used in all western blot experiments for the detection of SAP05. The OptimAb HA.11 monoclonal antibody (Eurogentec) was used to detect hemagglutinin (HA)-fusion proteins at the concentration of 0.5 μg/ml. The ANTI-FLAG monoclonal antibody (Sigma, F-3165) was used to detect FLAG tag-fusion proteins at a 1: 5000 dilution. Rabbit polyclonal anti-GFP antibody (Santa Cruz Biotechnology) was used with 1:10,000 dilution. Protein loading was visualized using Amido black staining solution (Sigma).

#### Co-expression assays in N. benthamiana leaves

Agroinfiltration-based transient gene expression in *N. benthamiana* leaves and co-immunoprecipitations were performed as described with minor modifications (MacLean et al., 2014). Briefly, 3XHA-TFs and SAP05 or GFP were expressed in *N. benthamiana* leaves for checking protein abundance. 3XHA-TFs and GFP-SAP05 or GFP constructs were expressed in *N. benthamiana* leaves for co-immunoprecipitation assays. The subcellular localization of GFP or RFP-tagged proteins transiently expressed in *N. benthamiana* leaves was visualized with Zeiss LSM 780 confocal microscope with the objective EC Plan-Neofluar 20x/NA 0.5. Images were taken with ZEN 2012 SP5 (Black) software and visualized with the ImageJ Fiji software.

#### GUS staining and real-time PCR

The expression of *rSPL11-GUS* and *rSPL13-GUS* reporter genes and the GUS staining of healthy or phytoplasma-infected plants at 4 weeks after phytoplasma inoculation was performed as described previously (Xu et al., 2016). The GUS-stained area on *A. thaliana* leaves was quantified with the ImageJ Fiji software.

#### *In vitro* binding assays

DNA that code for SAP05 (Ala33-Lys135), ZnF_AtSPL5 (Ser60-Leu127), SPL5 (full length), vWA domian of AtRPN10 (Val2-Gly193) or a vWA mutant (38GA39- > HS) were subcloned to either pOPINF (for N-terminal His tag), pOPINA (no tag or C-terminal His tag), pOPINS3C (N-terminal His-SUMO tag) or pOPINM (N-terminal His-MBP tag) (Berrow et al., 2007). The MBP-vWA fragment was amplified from pOPINM-vWA and ligated to PopinA to remove the His tag. The vectors were transformed or co-transformed into *E.coli* strain BL21 (DE3). Protein expression and affinity purification using immobilized metal affinity chromatography (IMAC) were carried out according to manufacture’s instruction (Ni-NTA agarose, QIAGEN). Briefly, protein expression was induced by the addition of 1 mM Isopropyl-β-D-thiogalactoside (IPTG) at 16°C for 20 h with shaking at 220 rev min^-1^. Cell pellets were lysed in IMAC buffer (50 mM Tris-HCl, 50 mM glycine, 0.5 M NaCl, 20 mM imidazole, 5% glycerol, pH 8.0) for affinity purification and eluted with elution buffer (50 mM Tris-HCl, 50 mM glycine, 0.5 M NaCl, 0.5 M imidazole, 5% glycerol, pH 8.0). Further purification was achieved by gel filtration (ÄKTA™ avant chromatography system) in gel filtration buffer (20 mM HEPES, 0.15 M NaCl, pH 7.5). When necessary, the tags were removed by HRV 3C protease in gel filtration buffer. For purifying the SAP05-vWA complex, MBP-vWA was co-expressed with His-SAP05. For testing complex formation in gel filtration, equal amount (molecular weight) of proteins were mixed in gel filtration buffer and left on ice for 45 mins before sample injection. For *in vitro* pull-down assay, His-tagged vWA or evWA domains were bound to 50 μL Ni-NTA agarose beads during protein purification starting from 50 mL cell culture and the beads were washed sequencially with IMAC buffer and gel filtration buffer. 20 μM SAP05 and/or ZnF domain of AtSPL5 were incubated with the Ni-NTA agarose beads in 100 μL gel filtration buffer for 1 h. After discarding the supernatant, the beads were washed sequencially with gel filtration buffer and IMAC buffer. Proteins bound to the beads were eluted in 100 μL elution buffer. Input samples and pull-down samples were analyzed by SDS-PAGE and Coomassie staining.

#### Degradation assay in human 26S proteasomes

For *in vitro* 26S proteasome degradation assay, highly purified human 26S proteasome preparation (BostonBiochem) was used immediately after thawing. 2.5 μg His-SPL5 and 5 μg SAP05 or 10 μg SAP05-vWA complex were added to 2 μg of 26S proteasome in 200 μL reaction buffer (50 mM Tris-HCl (pH 7.5), 50 mM Nacl, 10 mM MgCl2, 10% glycerol, 2 mM DTT, 5 mM ATP) and incubated at 28°C. 50 μM MG132 was added to inactivate the 26S proteasome activity. 20 μL aliquots from each reaction were collected at indicated times. Collected samples were added with SDS-PAGE loading buffer, boiled immediately and stored at −20°C until used for western blot analysis. Penta-His antobody (QIAGEN) was used at a 1:5,000 dilution for detecting His-fusion proteins. Recombinant vWA domain was probed with an anti-AtRPN10 polyclonal antibody (Agrisera) at 1:5000.

#### Phytoplasma infection assays

For *A. thaliana* inoculation, one leaf from a 4-week-old plant grown under short-day conditions was exposed to two or three AY-WB-carrier leafhoppers in a clip cage for 2 days. The leaf and the clip cage with carrier insects were then removed. For disease symptom recording, healthy or phytoplasma-exposed plants were kept in short-day conditions (10 h light/14 h dark, 22°C) for observing leaf development and survival or transferred to long-day conditions (16 h light/8 h dark, 22°C) for examining branching phenotypes.

#### Phylogenetic analysis

Phylogenetic analysis was performed on Phylogeny.fr web server (http://www.phylogeny.fr/index.cgi; Dereeper et al., 2008). Briefly, sequences were aligned with MUSCLE (v3.8.31) configured for highest accuracy. Phylogenetic trees were reconstructed using the maximum-likelihood method implemented in the PhyML program (v3.1/3,0 aLRT). Graphical representation and editing of the phylogenetic trees were performed with TreeDyn (v198.3).

### Quantification and statistical analysis

Statistical analysis was performed in Prism 7. One-way ANOVA was used to analyze experimental data with more than 2 two experimental groups followed by Tukey’s multiple comparisons test, and two-tailed unpaired Student’s t test was used for other data analysis.

## Data Availability

This study did not generate new dataset or code. Any additional information required to reanalyze the data reported in this paper is available from the lead contact upon request.

## Supplemental information

Document S1. Tables S3 and S4, related to Figures 2 and 4

Table S1. SAP05_Hybrigenics Y2H screen result, related to Figure 2

Table S2. SAP05_TF collection Y2H screen result, related to Figure 2

Table S5. Plasmids used in the study, related to STAR Methods
