# Supplementary material for: Parasitic modulation of host development by ubiquitin-independent protein degradation
Source: Cell. 2021 Sep 30;184(20):5201–5214.e12. doi: 10.1016/j.cell.2021.08.029 (PMC8525514; doi:10.1016/j.cell.2021.08.029)
Supplement: Document S1. Tables S3 and S4, related to Figures 2 and 4 [file mmc1.pdf]

Cell, Volume *184*

Supplemental information

Parasitic modulation of host development by ubiquitin-independent protein degradation

Weijie Huang, Allyson M. MacLean, Akiko Sugio, Abbas Maqbool, Marco Busscher, Shu-Ting Cho, Sophien Kamoun, Chih-Horng Kuo, Richard G.H. Immink, and Saskia A. Hogenhout

Supplemental tables

Table S3: Summary of Y2H analysis of SAP05 interactions with GATA or SPL transcription factors, Related to Figure 2

Gene name	Locus ID	Hybrigenics screening	*A. thaliana* TF library screening	Matchmaker gold Y2H system[a]	DUALhybrid Y2H system[b]
GATA1	At3G24050	+	-	+	NT
GATA2	At2G45050		-	+	NT
GATA3	At4G34680		-	+	NT
GATA4	At3G60530	+	-	+	NT
GATA5	At5G66320	+	-	+	NT
GATA6	At3G51080		-	+	NT
GATA7	At4G36240	+	-	+	NT
GATA8	At3G54810	+	-	+	NT
GATA9	At4G32890	+	-	+	NT
GATA10	At1G08000		-	+	NT
GATA11	At1G08010		-	+	NT
GATA12	At5G25830		-	+	NT
GATA13	At2G28340		-	+	NT
GATA14	At3G45170		-	+	NT
GATA15	At3G06740		-	+	NT
GATA16	At5G49300		-	+	NT
GATA17	At3G16870		-	+	NT
GATA18 (HAN/MNP)	At3G50870	+	+	+	NT
GATA19	At4G36620	+	+	+	NT
GATA20	At2G18380		+	+	NT
GATA21 (GNC)	At5G56860			+	NT
GATA22 (GNL)	At4G26150	+	-	-	NT
GATA23	At5G26930		-	+	NT
GATA24	At3G21175		-	+	NT
GATA25	At4G24470		+	+	NT
GATA26	At4G17570		+	NT	NT
GATA27	At5G47140	+	+	+	NT
GATA28	At1G51600		+	+	NT
SPL1	At2G47070		-	+	NT
SPL2	At5G43270		-	-	A.A.
SPL3	At2G33810		-	-	+
SPL4	At1G53160		-	-	+
SPL5	At3G15270		-	-	+
SPL6	At1G69170		+	+	NT
SPL7	At5G18830		-	-	-
SPL8	At1G02065		+	-	+
SPL9	At2G42200		+	-	A.A.
SPL10	At1G27370		-	-	A.A.
SPL11	At1G27360		-	+	NT
SPL12	At3G60030		-	+	NT
SPL13A	At5G50570		+	+	NT
SPL13B	At5G50670	+	+	+	NT
SPL14	At1G20980	+	-	+	-
SPL15	At3G57920		+	+	NT
SPL16	At1G76580		-	NT	NT

‘+’ indicates yeast growth; ‘–’ indicates no yeast growth; NT, non-tested; A.A., autoactivation.

[a]SAP05(33-135) was cloned into the pGBKT7 plasmid, and GATA or SPL transcription factors into the pGADT7 plasmid. Yeast strain AH109 was used for transformation and growth analysis.

[b]SAP05(33-135) was cloned into the pGADHA plasmid, and GATA or SPL transcription factors into the pLEXA-C plasmid as N-terminal fusions. Yeast strain NMY51 was used for transformation and growth analysis.

Table S4: Sequences of codon optimized SAP05 genes for generating transgenic plants, Related to figure 4

Gene	Codon-optimized sequence for plant transformation
SAP05_AYWB	ATGGCCCCGAATGAAGAGTTTGTGGGAGACATGCGAATAGTAAATGTCAAT<br>CTAAGTAATATAGATATACTAAAGAAGCATGAGACGTTTAAGAAGTACTTCG<br>ATTTTACACTCACGGGCCCCAGGTACAATGGCAATATAGCCGAATTTGCGA<br>TGATCTGGAAAATCAAAAACCCACCACTCAACCTCCTTGGGGTCTTTTTTGA<br>TGATGGTACGCGAGACGACGAAGATGACAAATACATTCTAGAGGAGTTGAA<br>ACAGATCGGCAACGGTGCAAAGAACATGTATATATTCTGGCAATATGAGCA<br>AAAGTAA
SAP05_WBDLa	ATGGCCCCTAACGAGGAGTTCGTTGGCAATATGAGGATCGTTAACATAACA<br>GTTTCAAATATAAACATCCTGAAGAATCACGCTACGTTCAAGCAATACTTTG<br>ACTTTAAGATCAACCGACCCTGCTATAATGGCAATATCGCAACCTTTGCCAT<br>AATGTGGAAGATAAAGAATCCTCCAAGAAACTTGTTGGGAGTTTTCTTTGAC<br>AATGGCACTAGGGATGATGAAGACGATAAGTATAACCTTGAGGACCTAAAG<br>AAAATGGGCAACGGAGCCTCAAATATGTATATCTTCTGGCAGTACGAACAG<br>AAGTAA
SAP05_WBDLb	ATGGCGCCCCCTCAGGATGAATTTATCAATGGAACCAGAATCGTGAACGTC<br>ATAGTAGCCTCAGGAGATATTTTGAAGAAACATAACTTATTTAAGCAATATTT<br>CGACTGGAGTTGCGAATACCCATCTTATAACAGCGAGTTGGAAGAGTTCGG<br>AATGATCTGGAAGATCAAAAACCCACCAGAAAACCTGCTTGGAGTTTTCTTC<br>GACGCAGGAAACCGTGACGACGCTGACAACAAGTATACCCTAGAGGAATT<br>GAAGTACATAGCTAATAAAGCCAAAAATATGTACATTTTTTGGCGTTACAAG<br>GAGAAGTAA
SAP05_PnWBa	ATGGCGCCTAGCCAGGAAGAAATTATCCAAGGGACTAGGATAGTCAATGTT<br>ACAGTCAGTAACATAAATGTTCTCAAGACCCACCCGAGTTTCGCACAGTATT<br>TTGATTTCAATCAGACTTGCCCATGCTATAATAGCACTGTAGCTGAGTTTTG<br>CATTATGTGGAAAATAAAGAACCCGCCCACAAATCTACTCGGAGTCTTCTTC<br>GATGAGAGCACCAGGGACGATGAAGATGACAAATACTCCCTAGAAGAACTT<br>AAGTATATGGCCAATAACTCCGTTAATATGTTCATATTCTGGGAACATAAGG<br>AAAAGTAA
SAP05_PnWBb	ATGGCTATGCCACCTAGAGAAGAGTTTATTGGTCAGACCAGAATAGTACAC<br>GTATCCATAGGAAACATAAATATCTTAAAGCAGCATGCTATTTTCAATAAGTA<br>CTTTGATTGGAGCTTGCAGTCAGCGCGTTACAACGAAGACTTGGAAGACTT<br>TTCAATGATCTGGACAATAAAAGATCCGGACCCGAATCTGCTAGGGGTTTT<br>CTTCGACGGTGGAATCAGACATGGACAAGACGACACATACAACCTACAGGA<br>GCTCAAACACATGGGGAATGGGGCCAACAATATGTACTGCATATTTCTGAA<br>GAACAATTAA
